# Daytime and seasonal reflectance of maize grown in varying compass directions

**DOI:** 10.3389/fpls.2022.1029612

**Published:** 2022-09-23

**Authors:** Claudia Buchhart, Urs Schmidhalter

**Affiliations:** ^1^ Chair of Restoration Ecology, Department of Life Science Systems, Technical University of Munich, Freising, Germany; ^2^ Chair of Plant Nutrition, Department of Life Science Systems, Technical University of Munich, Freising, Germany

**Keywords:** corn, drone, flight time, multispectral reflectance, row crop, row orientation

## Abstract

High temporal and spatial resolution is required to meet the challenges of changing plant characteristics over time. Solar radiation and reflectance of vegetation canopies vary with the time of day and growing season. Little is known regarding the interactions between daily and seasonally varying irradiation and reflectance of row-planted crops that can be grown in any compass direction. The spectral reflectance of maize grown in four compass directions was recorded across the entire life cycle through highly frequent drone-based multispectral sensing to determine biomass changes over time and make early yield predictions. Comparison of information from spectral bands and indices indicated no differences among the four compass directions at the reproductive stage and only a few differences at the earlier vegetative growth stages. There was no systematic influence of row orientation on the relationships between spectral data, biomass, and grain yield, except at the early growth stages. Spectral relationships to biomass at the reproductive stage varied in row directions with R^2^-values close to 0.9, already observed at early growth stages for the indices NDVI, SR, GCI, and GNDVI. The spectral relationships to yield were closer in individual compass directions, with R^2^-values varying between 0.8–0.9 for the best indices GCI and GNDV after BBCH 61. A closer inspection of daytime changes indicated a diurnal trend with 15 and 20% decreased spectral values observed after midday at the growth stages BBCH 81 and 61, respectively, thus requiring standardization of flight timing during the day. Drone-assisted nadir-oriented spectral sensing could be a reference for terrestrial and satellite-based reflectance sensing to relate canopy reflectance to crop characteristics quantitatively.

## Introduction

In the last decade, unmanned drones, which can be used flexibly, efficiently, and cost-effectively and have a spatial and temporal resolution previously unsurpassed, have been increasingly used in agricultural applications (Jin et al., 2021). Possible applications arise in precision farming, optimizing resource inputs for sowing, fertilization, and crop protection by adapting land use management to small-scale heterogeneity (Schmidhalter et al., 2008). High-throughput phenotyping by drones is increasingly being used in breeding to evaluate the complex plant traits of large numbers of lines (Hu et al., 2020). These methods allow optimized, resource-efficient management and higher-performing crop selection.

Together with biophysical parameters, crop growth varies over time and space; therefore, remote sensing remains challenging because of the complex nature of plants (Marais-Sicre et al., 2014; Huang et al. (2021)). Advances in monitoring vegetation using remote sensing critically depend on quantitatively relating canopy reflectance to crop characteristics (Goel and Grier, 1987).

Information obtained by remote sensing is influenced by many factors, such as cropping patterns. For example, row crops differ from broadly sown crops, changes in plant geometry, lineages of crops in compass directions, biomass and nutritional status, and varying irradiation intensities and angles. Therefore, spectral sensing with high temporal and spatial resolution is required to address the challenge of changing plant characteristics over time. This is difficult to perform with high-clearance sensor-equipped vehicles, considering the time requirements in large fields (Mistele and Schmidhalter, 2008), even more, when done manually because of the limited accessibility of tall-growing crops. In addition, the relatively small measurement area (one row at most) of handheld sensors is linked with the potential for high variability caused by interrow differences. Alternatively, satellite-based surveys cannot provide the temporal and spatial high-resolution information needed for the required precision.

An excellent alternative is provided by multispectral unmanned aerial vehicle (UAV) sensing. Its potential has rarely been determined across the entire life cycle of plants to determine biomass changes over time and make early yield predictions. Although the first studies on diurnal and seasonal influences on the spectral signature of drones have been reported for wheat (De Souza et al., 2021), such studies are lacking for maize, which is one of the most important crops grown worldwide. This requires highly managed experimental designs to determine the possible daytime and seasonal influences of varying solar radiation and angles on the spectral signatures of maize grown in various compass directions.

A close match between the spectral footprint and destructively assessed areas for referencing biomass or yield is relevant. Simplified referencing with a small number of plants is generally insufficient because of heterogeneity within maize stands. To preclude this, it has been suggested that at least 2.5 maize rows in the field of view should be measured (Major et al., 2003), or the length of the destructively assessed rows should be extended.

Typically, rows of plants are arranged parallel to the longer side of the field, implying that plant rows can have any possible orientation. Assuming that the sun never remains directly above the stands, a change in row orientation always causes a change in the top-of-canopy reflectance because the sunlit/sun-shaded and plant/soil fractions composing the reflectance signal change (Kuester and Spengler, 2018). Row crops with two-dimensional inhomogeneity are particularly challenging because of their spectral coverage and the influence of the row azimuthal direction (Goel and Grier, 1987), the share of mixed soil-plant pixel information, and shadows.

Solar radiation and the bidirectional reflectance characteristics of vegetation canopies vary with the time of day and through the growing season (Ranson et al., 1985; Li et al., 2021), influenced by solar radiation, temperature, and shade levels. As crops can be grown in any compass direction, depending on the orientation of the fields, this may affect productivity and spectral reflectance. Sowing direction can increase or reduce the interception of solar radiation by plant leaves, which changes the shading between plants in the rows (Correa et al., 2019). Karlen and Kasperbauer (1989) concluded that the sowing direction that led to the maize crop’s best results was north-south, with a spacing of 0.76 m between rows. The influence of crop row orientation has been demonstrated in several wavelength domains, and it is thus important to take row orientation into account in the physical or empirical methods used to improve the estimate of biophysical parameters of crops, such as biomass or yield (Marais-Sicre et al., 2014).

Most studies thus far have focused only on a single-row orientation, such as the NS orientation (Zarco-Tejada et al., 2005), and the coupled effects of row orientation and canopy reflectance are not well understood (Li et al., 2020). To the best of our knowledge, there has been no systematic field investigation on the diurnal and seasonal observation time for crop variable estimation from canopy reflectance spectra of row crops in multiple compass directions.

The timing of sensing can also affect the accuracy of yield prediction and nutrient need assessment (Maresma et al., 2020). Changes in reflectance during the day will impact NDVI results and the algorithms for predicting yield and crop responsiveness to nitrogen from such data (Raun et al., 2005). Final yield is important for evaluating the efficiency of field management practices and/or making adjustments over time (Long and Ketterings, 2016) and should be predicted earlier in the season. Unfortunately, little is known regarding the interactions between daily and seasonally varying irradiation, in combination with the cultivation of crops in different compass directions. This study fills this gap by recording frequent spectral signatures across the season and within the day with maize grown in four compass directions. Spectral reflectance was recorded in nadir orientation through drone-based multispectral sensing, relationships to biomass and yield were determined, and possible early yield predictions were assessed. For this purpose, maize was grown in two uniform experimental plots, with varied nitrogen fertilization in the compass directions N, NE, E, and SE, and overflown over 21 flights in the east-west direction during the season. At three time points, biomass was sampled in two 6-m long rows, and, at the end of the experiment, the yield was recorded in each of the four 6-m rows. The interrow variation in the spectral information was also assessed.

This study investigated the interactions between daily and seasonally varying irradiation and the reflectance of maize grown in four compass directions through highly frequent drone-based multispectral sensing. A better knowledge of these interactions should allow for better quantitative estimates of biomass and early yield predictions by spectral sensing, taking diurnal and seasonal changes into account.

## Material and methods

### Study site, experimental design, cultivation, and plant sampling

**Figure 1:**
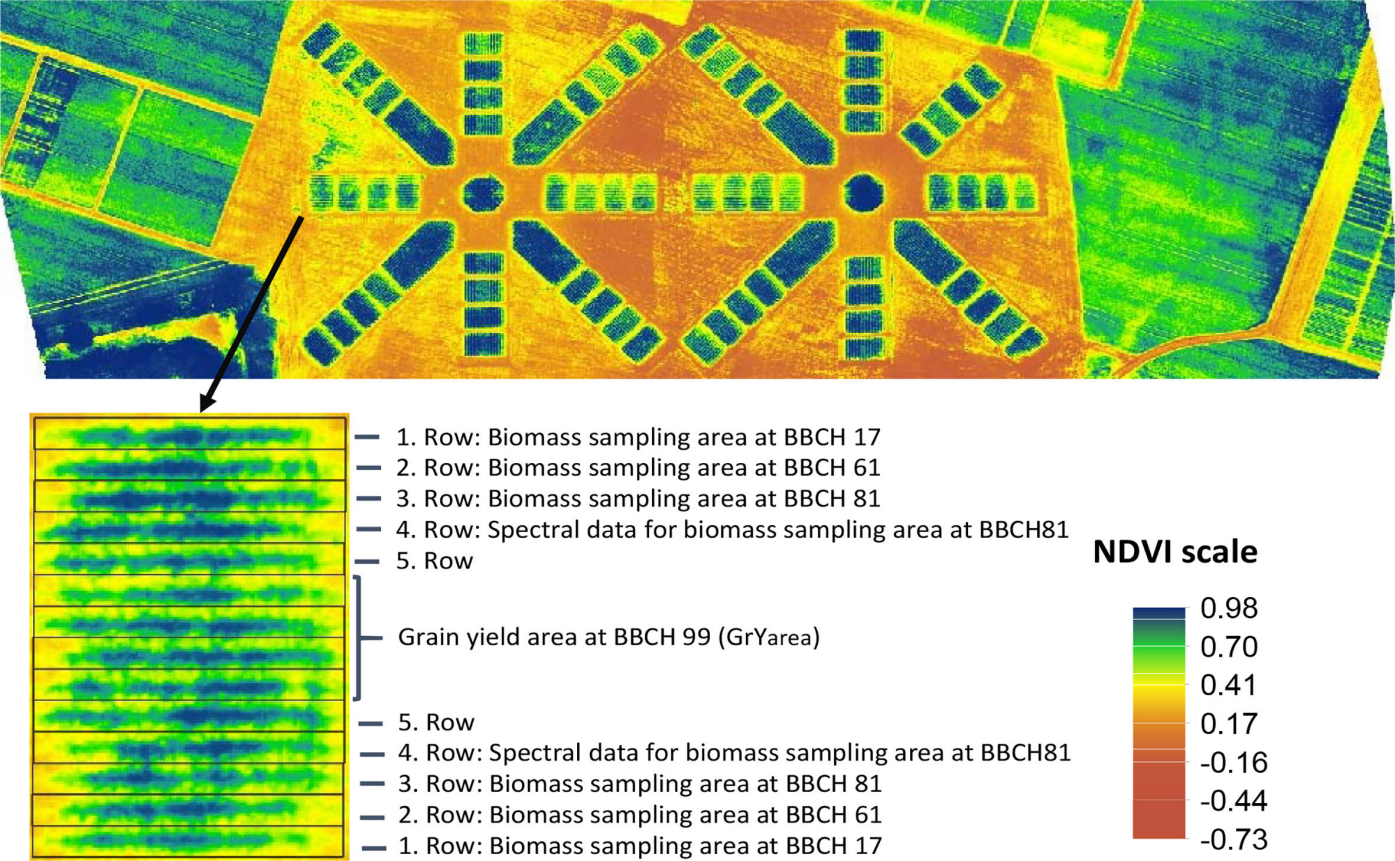
NDVI image of maize plots from July 03, 2019, at BBCH 17 at 9:15 am obtained with an eBee drone equipped with a Parrot Sequoia multispectral camera illustrating the plot design of the experiment resembling two stars. The detailed plot view indicates the rows used for the three destructive biomass samplings at BBCH 17, 61, and 81 and the rows used for grain yield harvesting at BBCH 99.

The trial was conducted in 2019 in Freising at the Dürnast Research Station of the Technical University of Munich, located in southwestern Germany (11°41’E; 48°24 N, 450 m asl). Precipitation averaged 688 mm, and the annual mean temperature was 9.8°C in 2019. Maize (*Zea mays* cv. LG 30258) was grown in two adjacent trials on a Cambisol consisting of silty clay loam in four different row orientations (compass direction north = N; northeast = NE; east = E; southeast = SE) with a row spacing of 0.75 m, a row length of 6 m per plot and 14 rows per plot. The experimental design, resembling two stars, is depicted in **Figure 1** and consists of four plots on opposite outer sites per row alignment, totaling 64 plots. Additionally, a central circle plot and three or four rectangular plots were cultivated on the inner sides of the left and right trials (**Figure 1**), respectively, which were not used in the analysis. After plant emergence, 9.5 plants per square meter were counted on average. Four nitrogen fertilization levels (0, 50, 80, and 160 kg/ha) were applied to the four plots on each of the two sides per row alignment. Owing to some damage caused by feral hogs and an error in fertilizer application, the number of replicates in the nitrogen fertilizer variants per alignment was not equal. Adequate amounts of P, K, Mg, and S were also supplied. Three destructive biomass samplings were conducted on July 3 (BBCH 17; seven leaves unfolded), July 23 (BBCH 61; ear tip emerged from leaf sheath), and August 28 (BBCH 81; early dough) 2019 (BBCH scale, according to Biologische Bundesanstalt, Bundessortenamt und Chemische Industrie), using the two opposite outer rows of each plot (on average 82 plants) with a green forage chopper fitted with a weighing unit. For grain yield determination, the middle four rows within the plots (GrYarea) were harvested using a combine plot harvester on October 17, 2019, at BBCH 99 (**Figure 1**).

### Aerial data collection and flight conditions

Twenty-two UAV flights were conducted for 13 days between June 27, and September 11, characterized by varying sun positions and different growth stages, using the multispectral camera Sequoia^+^ (Parrot, France) mounted on the wing aircraft eBee (senseFly, Lausanne, Switzerland). On three days, July 4 and 22, and August 27, at BBCH 17, 61, and 81, respectively, the trials were overflown several times to assess the daytime effects.

To determine soil coverage, the UAV was equipped with a red-green-blue SODA camera (senseFly, Lausanne) and overflown on June 27 and July 3, 16, and 22, 2019.

**Figure 2:**
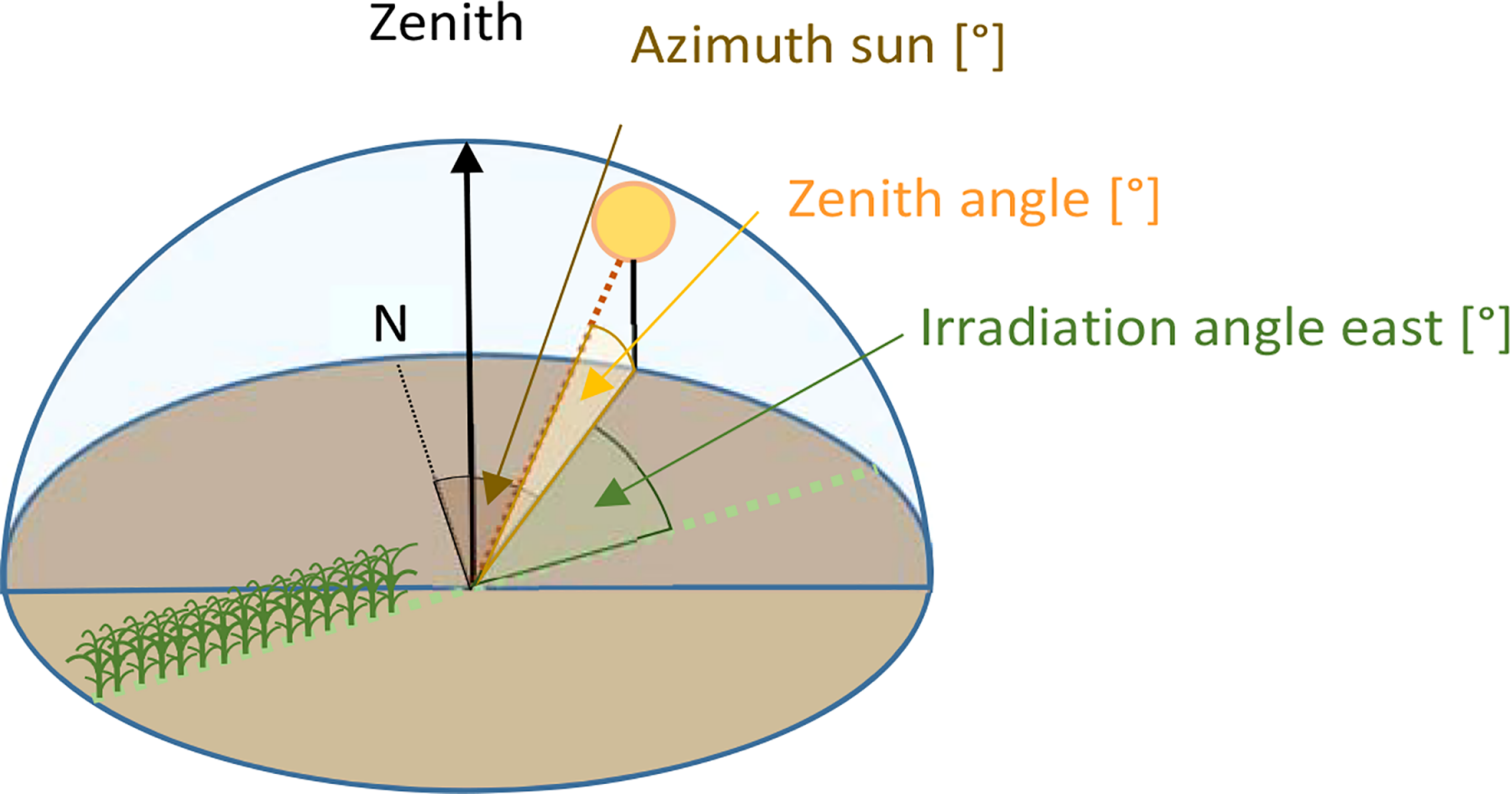
Sketch illustrating the geometry of the azimuth, zenith, and irradiation angle.

During most flights lasting 15 min, sunny weather conditions prevailed (**Table 1**). Climate data were obtained from the Climate Data Center of DWD. (Deutscher Wetterdienst, Germany). Climate data were obtained from a weather station 500 m away from the experimental site. The solar azimuth and zenith angle (**Figure 2**) were determined at mid-flight using information from the web page http://solartopo.com/sonnenumlaufbahn.htm to calculate the irradiation angle (**Appendix Figure 1**).

**Table 1:** Growth stages, solar azimuth, zenith angle, and weather conditions during the UAV flights.

Flight No	Date	Flight mid-time	BBCH scale	Solar azimuth [°]	Solar zenith angle [°]	Sum of incoming solar radiation [Wh/m²]	Sum of incoming solar radiation per hour [J/cm²]	Minutes of sunshine duration per h	Air temperature at 2 m height [°C]
1	27.6.19	11:00	16	121	53	874	315	60	29.3
2	4.7.19	9:15	17	96	36	790	284	60	20.9
3	4.7.19	13:35	17	189	64	812	292	60	24.5
4	4.7.19	16:04	17	247	48	455	164	60	25.4
5	5.7.19	10:26	17	252	45	904	326	60	23.8
6	16.7.19	9:54	55	105	41	787	283	48	20.0
7	22.7.19	9:07	61	96	33	722	260	60	24.7
8	22.7.19	11:07	61	125	51	470	169	24	26.4
9	22.7.19	13:11	61	176	62	781	281	60	28.5
10	22.7.19	14:44	61	218	57	599	216	56	28.8
11	22.7.19	16:09	61	245	46	443	159	60	28.9
12	30.7.19	8:20	69	88	24	248	89	0	20.3
13	8.8.19	14:22	73	207	55	698	251	54	24.9
14	14.8.19	9:41	75	108	34	738	266	54	18.8
15	22.8.19	13:10	79	178	53	708	255	57	20.4
16	27.8.19	9:14	81	106	27	633	228	60	23.3
17	27.8.19	11:09	81	135	44	764	275	57	25.7
18	27.8.19	14:23	81	206	49	512	184	60	27.6
19	27.8.19	15:54	81	234	39	278	100	60	27.5
20	4.9.19	15:29	85	226	40	337	121	60	24.0
21	10.9.19	13:57	89	197	45	518	187	30	17.9
22	11.9.19	9:46	89	118	29	713	257	60	17.0

### Spectral reflectance indices and aerial data processing

The canopy reflectance of four spectral bands (green 550 nm ± 20 nm; red 660 nm ± 20 nm, red edge 735 nm ±5 nm, and NIR 790 nm ± 20 nm) were acquired at a flight altitude of 50–60 m above ground level, resulting in a resolution of approximately 6 cm/pixel. For mission planning, eMotion 3 and Pix4D software from senseFly (Lausanne, Switzerland) were used to provide an 85% lateral and 95% longitudinal overlap. Further details were reported by De Souza et al. (2021). Calibration was performed using a white reference standard to process UAV data for each flight. Pixel mean values and standard deviations within single plot rows were extracted from the UAV images for each band using ArcMap Version 10.5 (Esri) and batch-processed with the ArcPy geoprocessing library package of the Python programming language.

Based on the image from July 3, a signature file was created to generate with maximum likelihood classification (MLC) a classified raster (soil and plant) in ArcMap Version 10.5 (Esri) as output. The extracted histogram was used to determine the soil coverage.

Spectral relationships were established for the biomass assessed at BBCH 81 and grain yield at BBCH 99. For this purpose, previously used spectral indices enabling a good correlation with biomass and grain yield (**Table 2**) (De Souza et al., 2021) were calculated for the extracted spectral mean values of the respective plot rows (**Figure 1**). Spectral indices from the fourth plot row were used to establish spectral relationships to the destructively assessed biomass yield at BBCH 81, as the third row was a border row after biomass sampling on July 23. The biomass at BBCH 17 and 61 were not evaluated using spectral data.

**Table 2:** Spectral reflectance indices and the respective equations. Spectral reflectance is indicated by the letter R.

Index name	Equations	Reference
Normalized difference vegetation index	NDVI = (R790-R660)/(R790+R660)	[Rouse et al., 1974]
Green normalized difference vegetation index	GNDVI = (R790-R550)/(R790+R550)	[Ma et al., 1996]
Simple ratio	SR = R790/R660	[Pearson and Miller, 1972]
Green chlorophyll index	GCI = (R790/R550)-1	[Gitelson et al., 2003]
Red-edge chlorophyll index	RECI = (R790/R735)-1	[Gitelson et al., 2005]
Normalized difference red-edge index	NDREI = (R735-R550)/(R735+R550)	[Hassan et al., 2018]

### Statistical analysis

Analysis of variance (ANOVA) followed by Tukey’s test (P < 0.05) was used to determine the differences between the three destructive biomass samplings and grain yield within the row orientations. The non-parametric Kruskal–Wallis test was performed, followed by the Dunn-Bonferroni *post-hoc* test, to test for significant pairwise differences (P < 0.05) among row orientations, comparing different spectral bands and indices. All statistical tests, Levene’s test of equality of error variances, and the test for normal distribution were conducted using IBM SPSS statistics 28. Pearson correlation coefficients, coefficients of variation, and root mean square errors (RMSEs) were calculated using Microsoft Excel (Microsoft Office Professional Plus, 2016). The daytime effects of the indices, comparing measurements at the same growth stage, were tested usinga repeated-measures ANOVA with a Greenhouse-Geisser correction.

## Results

### Soil coverage, biomass, and grain yield

**Figure 3:**
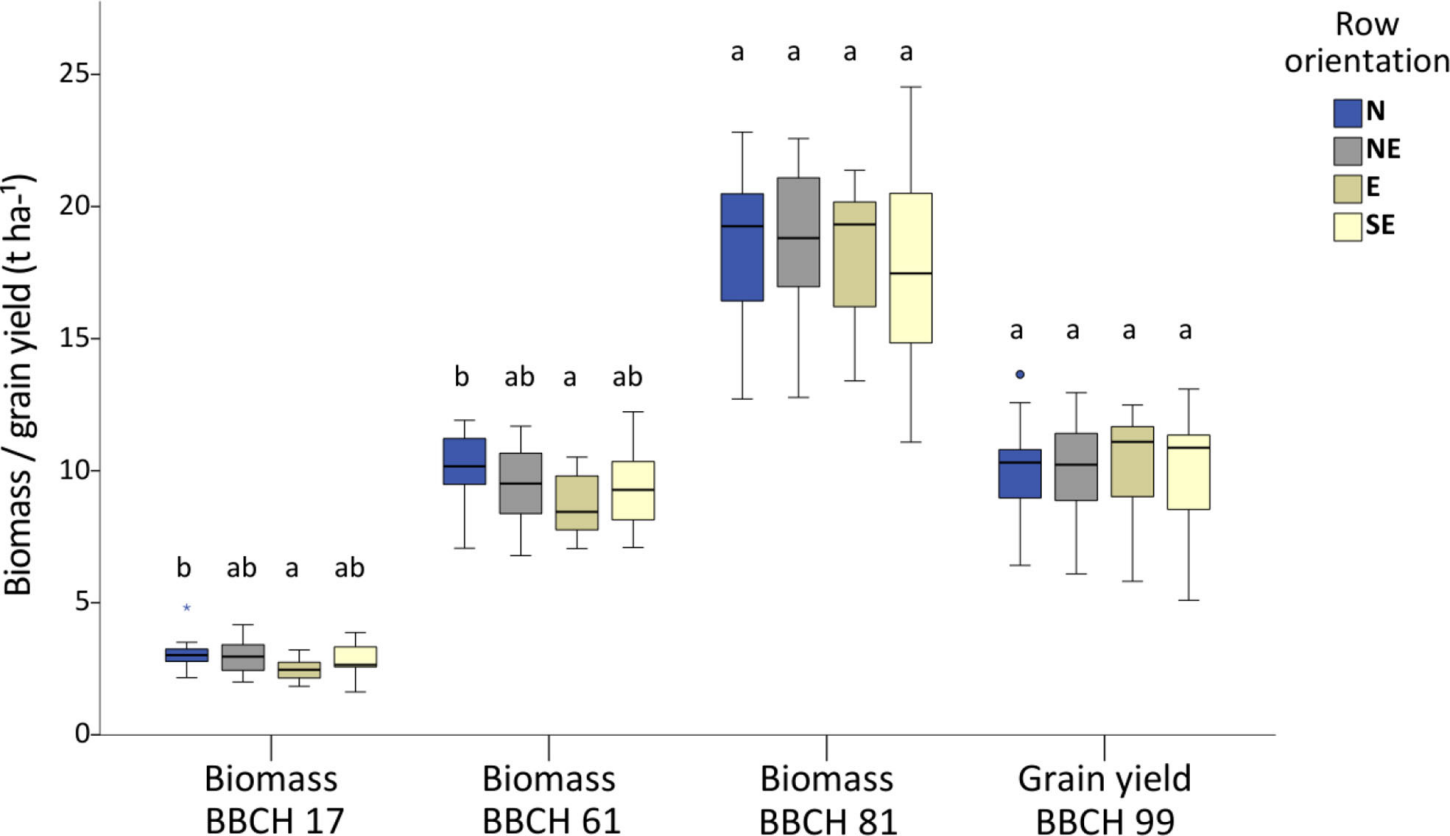
Boxplots of the three destructive biomass samplings and the final grain yield in row orientations at the respective growth stages (BBCH). Statistical differences are indicated at P < 0.05 with different letters.

Soil coverage was 31% at BBCH 16, increasing rapidly to 59% at BBCH 17, 70% at BBCH 55, and 73% at BBCH 81. The biomass of the first sampling at BBCH 17 averaged 2.8 t ha^-1^, increased to 9.4 t ha^-1^ in the second sampling at BBCH 61, and increased to 18.3 t ha^-1^ in the third sampling at BBCH 81. Grain yield averaged 10.1 t ha^-1^, indicating a standard deviation of 2.0 t ha^-1^, ranging from 5.1 to 13.65 t ha^-1^ (**Figure 3**; **Table A1**).

Biomass yield from the first sampling was related to the second and third sampling with a Pearson coefficient of determination of R² = 0.54 and 0.31, respectively, whereas biomass from the second sampling was related to the third sampling with R² = 0.56. The grain and biomass yields of the three consecutive samples were related to each other with R² = 0.32, 0.53, and 0.74, respectively. Yield relationships also varied for different row orientations (**Table A2**). The east direction exhibited a low R² value of 0.12 for the biomass assessment at BBCH 17 and 81.

ANOVA investigating the influence of row orientations indicated a significant difference in the first and second biomass sampling at BBCH 17 and BBCH 61, respectively, with P-values of 0.01 and 0.03. In contrast, no differences were observed in the third biomass sampling at BBCH 81 and the grain yield harvest at BBCH 99. The differences were due to the lower yield in the east orientation and the higher yield in the north orientation which differed by about 1. In the east orientation, the standard deviation and range were lower than those for the other orientations in all biomass samplings at BBCH 17, 61, and 81, but not in the grain yield assessment at BBCH 99 (**Table A1**).

### Spectral comparison between rows of the same orientation, bands, and indices

Spectral information between rows of the same orientation was closely related. The indices of the third, fourth, and fifth rows closely matched those of the four inner plot rows representing the grain yield area (GrYarea), with an average R² value of 0.94 across all measurements (**Figure A2**). Comparing the bands within each measurement, green had, on average, the most robust agreement, with red and red edges having R² values of 0.73–0.74. NIR showed the weakest agreement, with all other bands having R² values of 0.09–0.19. Among the indices, GCI and GNDVI with R² = 0.97–0.99 and NDVI and SR with R² = 0.92–0.98 were also closely related. NDREI and RECI showed the weakest relationship, with R² = 0.5 (**Appendix Table 3**).

### Spectral pairwise comparison of different row orientations

Pairwise comparison of spectral bands or indices between different row orientations in the grain yield area showed no significant differences (flights 5, 6, 8, and 10 –22) or only a few differences (flights 4 and 9) after BBCH 17 (**Table 3**). In contrast, differences were consistently observed on flights 1 and 2 and often on flights 3 and 7. In most cases, the row orientation toward the east contributed to the significant differences associated with significantly lower biomass until BBCH 61 (**Figure 3**).

**Table 3:** Spectral differences between row orientations using the Kruskal–Wallis test depicting significant or non-significant (n.s.) differences.

Flight	BBCH	Green	Red	Red edge	NIR	GCI	GNDVI	RECI	NDVI	SR	NDREI
**1**	**16**	0.01* (2,4,6)	0.02* (2,4,6)	0.01* (2,4,6)	0.02* (4,6)	0.03* (2,4)	0.03* (2,4)	0.04* (2,4)	0.04* (2,4)	0.02* (2,4)	0.04* (2,4)
**2**	**17**	0.00* (2,4,6)	0.00* (2,4,6)	0.03* (2,3.4)	0.01* (4,6)	0.00* (2,4,6)	0.00* (2,4,6)	0.00* (2,4,6)	0.00* (2,4,6)	0.00* (2,4,6)	0.00* (2,4,6)
**3**	**17**	n.s.	0.04* (1,4)	0.04* (1,4)	0.01* (1,4,6)	n.s.	n.s.	n.s.	n.s.	n.s.	n.s.
**4**	**17**	n.s.	0.04* (2,6)	n.s.	n.s.	n.s.	n.s.	n.s.	n.s.	n.s.	n.s.
**5, 6**	**17-55**	n.s.	n.s.	n.s.	n.s.	n.s.	n.s.	n.s.	n.s.	n.s.	n.s.
**7**	**61**	n.s.	0.01* (1,4)	n.s.	n.s.	n.s.	n.s.	n.s.	0.01* (1,4,6)	0.00* (1,4,6)	0.00* (2,4,6)
**8**	**61**	n.s.	n.s.	n.s.	n.s.	n.s.	n.s.	n.s.	n.s.	n.s.	n.s.
**9**	**61**	0.02* (4,6)	n.s.	n.s.	n.s.	n.s.	n.s.	n.s.	n.s.	n.s.	0.05* (6)
**10 - 22**	**61-89**	n.s.	n.s.	n.s.	n.s.	n.s.	n.s.	n.s.	n.s.	n.s.	n.s.

Significant differences in the pairwise comparison of row orientations using the posthoc test are indicated with p < 0.05 in brackets, and were 1 = N-NE, 2 = N-E, 3 = N-SE, 4 = NE-E, 5 = NE-SE, 6 = SE-E. * indicates the significance level at p < 0.05.

### Index performance in individual row orientations as related to biomass yield at BBCH 81 and grain yield at BBCH 91

**Figure 4:**
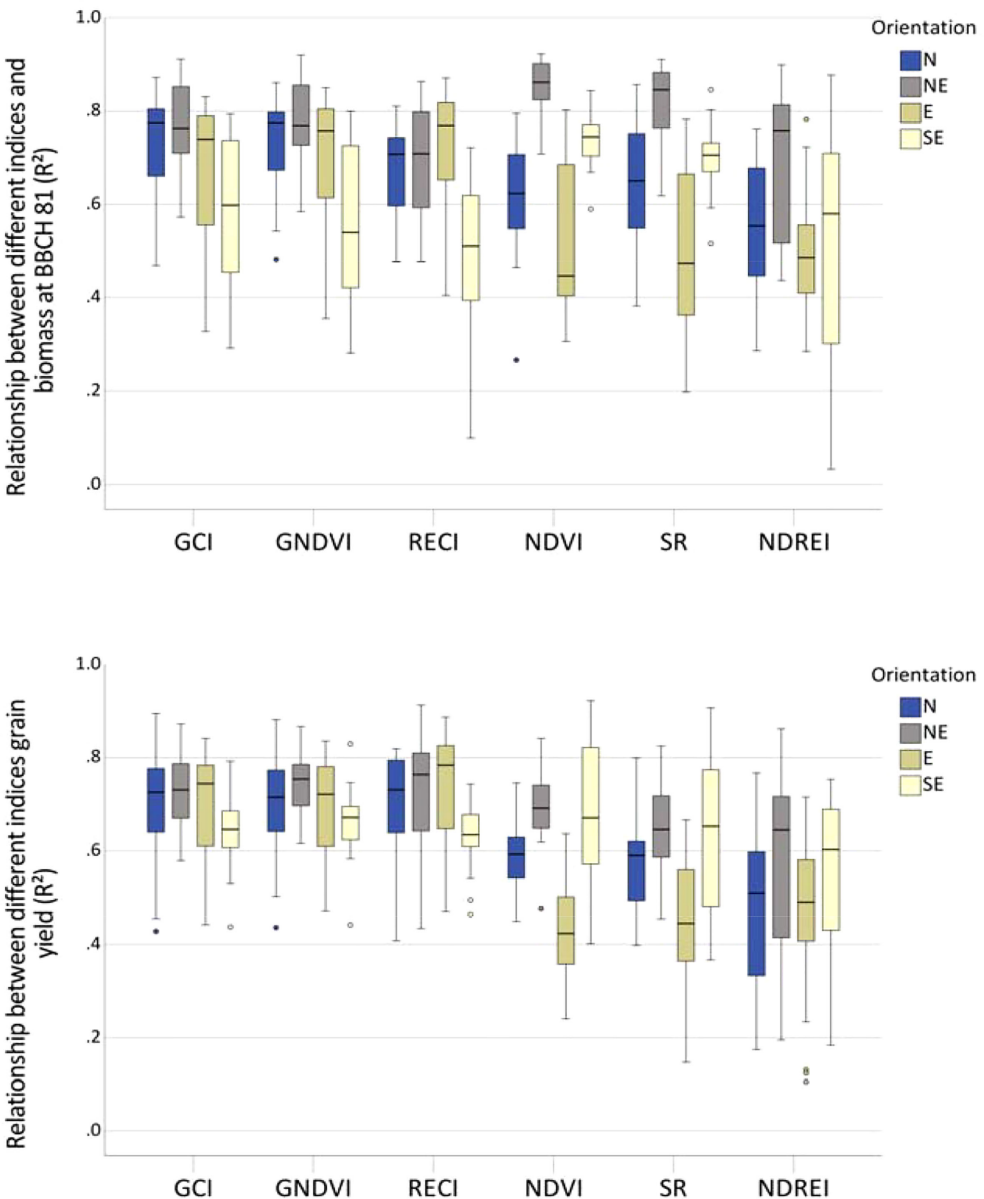
Boxplots depicting relationships between different indices and **(A)** biomass at BBCH 81 and **(B)** grain yield, in each row orientation, indicated as Pearson coefficients of determination across the season.

The closest relationship between the indices and biomass assessed at BBCH 81 in individual orientations (N = 16) was predominantly obtained in the NE orientation (**Figure 4A**). For the GCI, GNDVI, and NDVI, high R² values with 0.91–0.92 were already obtained at BBCH 16 and 17 (**Figure A3**). Relationships with biomass at BBCH 81 varied depending on the index, row orientation, and individual flights (**Figure A3**). Although all indices frequently reached an increased R² value of 0.8, only NDVI showed R² values > 0.7 in all row orientations at BBCH 73–81. The GCI and GNDVI performed best with R² = 0.69, compared to NDVI, SR, and RECI with R² = 0.68, 0.67, and 0.64, respectively. NDREI showed the lowest R² value of 0.56.

Relationships between indices and grain yield varied less among compass directions, particularly for the GCI and GNDVI (**Figure 4B**), providing the tightest relationship with R² values of up to 0.89 between BBCH 61 and 89. In contrast, at the early growth stage of BBCH 16, only weak relationships between the indices and grain yield were observed, except for the RECI in the NE orientation (**Figure A3**). The NDVI and SR were comparably related to grain yield as the GCI and GNDVI; however, only from BBCH 75 onwards delivered less stable information regarding row orientation. Across all flights, grain yield was best related to GNDVI, RECI, and GCI, with R² values of 0.70, 0.70, and 0.69, respectively, whereas NDVI, SR, and NDREI were significantly less related, with R² values of 0.59, 0.58, and 0.52, respectively.

### Performance of indices detecting biomass yield at BBCH 81 and grain yield at BBCH 99 across all row orientations

**Figure 5:**
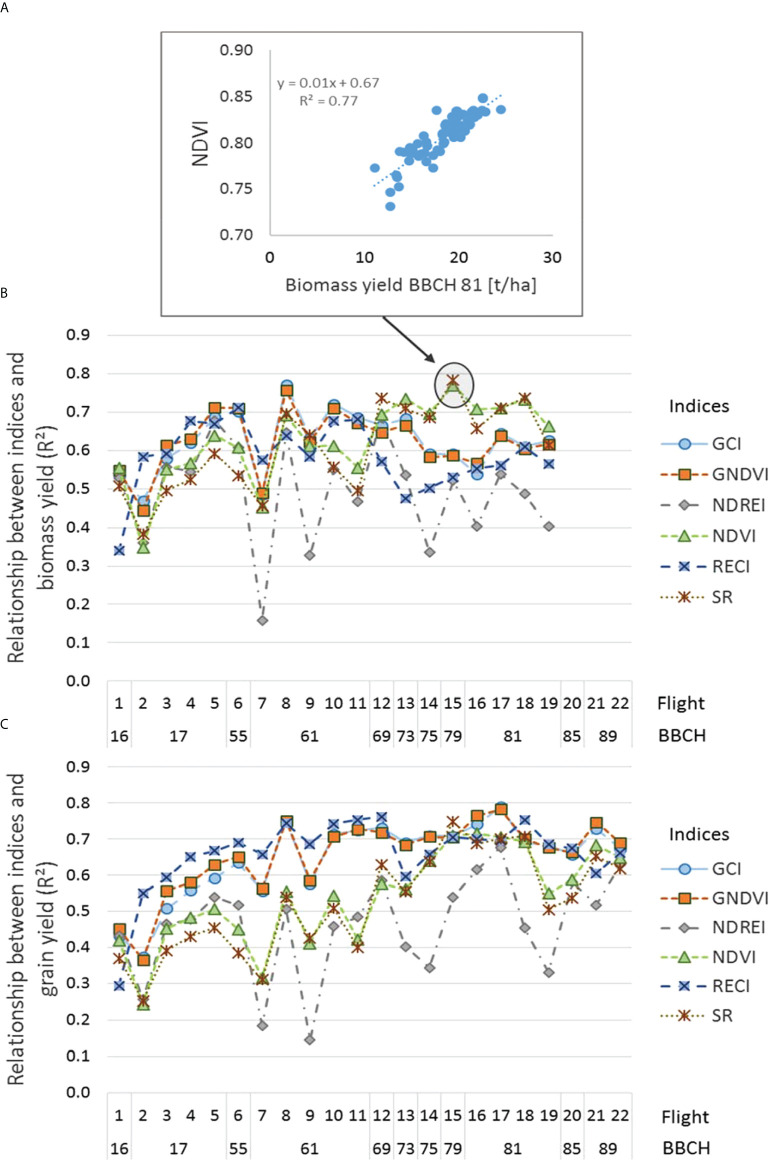
**(A)** Relationship between the NDVI determined at BBCH 79 and biomass assessed at BBCH 81; **(B)** Seasonal relationships between various indices and biomass at BBCH 81 indicated as Pearson coefficients of determination (R²); **(C)** Seasonal coefficients of determination (R²) between grain yield and various indices.

Across all row orientations (N=64), the biomass destructively determined at BBCH 81 was closely related to the SR, and the NDVI determined at BBCH 79 with R² values of 0.79 and 0.77, respectively (**Figures 5A**, **B**). Comparably high R² values were obtained with the GCI and GNDVI at BBCH 61, with R² values of 0.77 and 0.76. Starting at BBCH 17, the RECI, GCI, and GNDVI showed the closest relationships. However, the relationship to biomass was less tight for the RECI after BBCH 61, whereas after BBCH 69, the R² values were highest for NDVI and SR. The closest relationship to biomass at BBCH 81 was achieved by the NDVI, GNDVI, and GCI indices, with R² = 0.63, 0.62, and 0.62, respectively. Grain yield was best assessed using the GCI and GNDVI at BBCH 81, with R² values of 0.79 and 0.78 (**Figure 5C**). These indices have already been shown at BBCH 61, with particularly close and consistent relationships with biomass. Less strong relationships were observed for NDVI, SR, and NDREI, with R² values of 0.52, 0.52, and 0.46, respectively. The indices RECI, GCI, and GNDVI, achieved the closest and most consistent relationships with grain yield across the growing season, with R² values of 0.66, 0.65, and 0.65, respectively. They indicated that early in the growing season at BBCH 17, there were close relationships to grain yield, for example, for RECI having an R² of 0.67 with an average RMSE of 1.2 t ha^-1^ (**Table A4**).

### Seasonal variation of spectral indices

**Figure 6:**
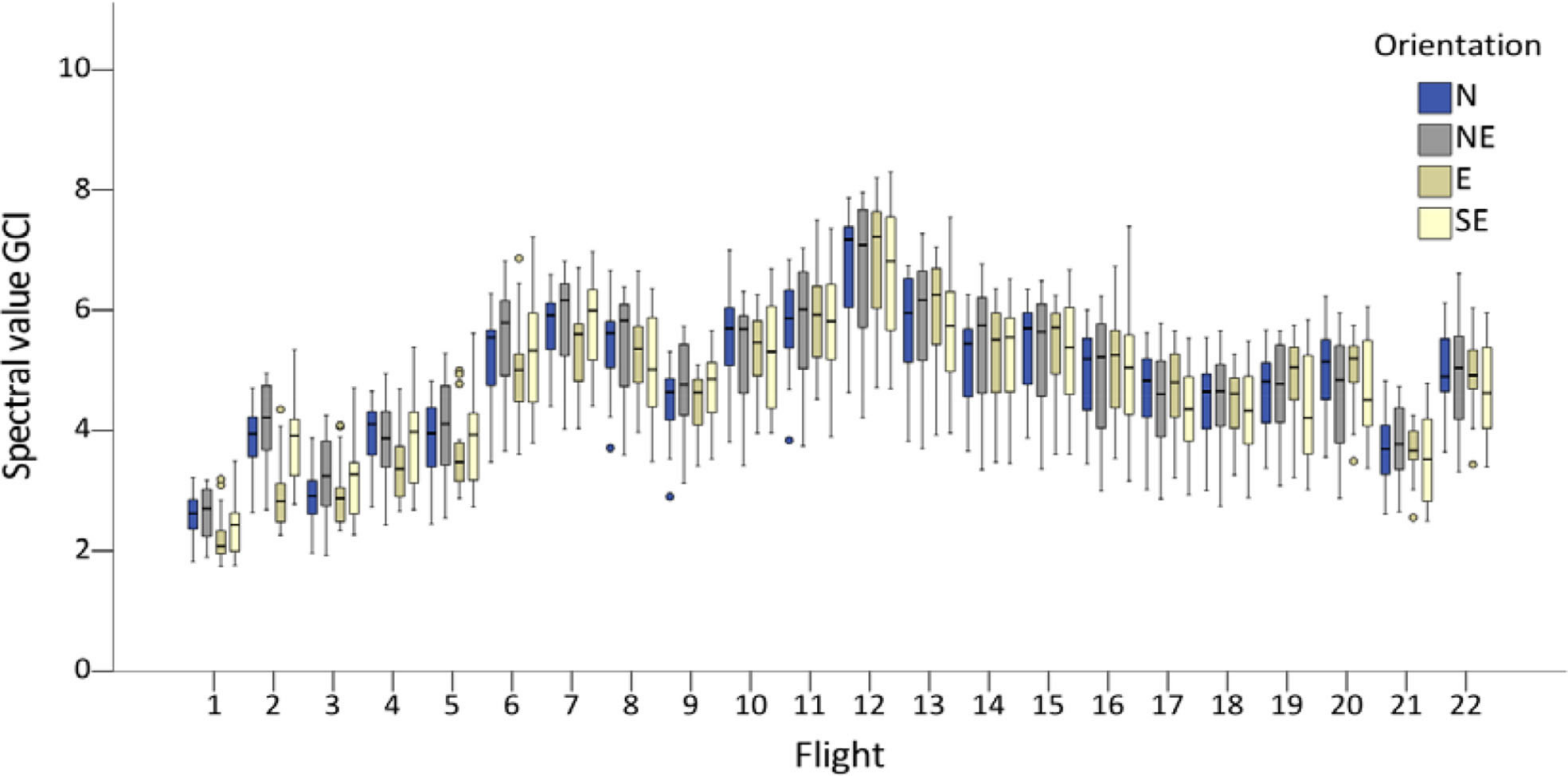
Boxplots of GCI values for each flight and row orientation (N = north, NE = northeast, E = east, SE = southeast). The boxes indicate repeated spectral measurements within the growth stages BBCH 17, 61, and 81.

Disregarding daytime changes, the spectral values generally increased until BBCH 69 (flight 12), as illustrated exemplary for the index GCI in **Figure 6**, and subsequently decreased. However, the spectral values differed substantially within the same growth stages, for example, at BBCH 61 (flights 7–11). Subtle differences were observed between BBCH 17 and 81. A repeated-measures ANOVA with a Greenhouse-Geisser correction indicated statistically significant differences within BBCH stages 17, 61, and 81, with p < 0.001 for all indices. Bonferroni-adjusted *post-hoc* analysis revealed significant differences (p < 0.001) between measurements at the same BBCH growth stage; however, no significant differences in row orientations were found (**Table 3**).

## Discussion

Biomass and grain yield did not differ in different row compass directions at the BBCH 81 and 99 reproductive growth stages (**Figure 3**). This allows for excluding row direction effects of biomass or grain yield in spectral comparisons. In addition, the only difference observed was caused by decreased biomass in the east direction compared to that in the north direction at the earlier vegetative growth stages at BBCH 17 and 61 (**Figure 3**). During the early growth stages, biomass differences in different row directions may contribute to different spectral information. Further discussion will focus on reproductive growth stages and address the differences observed in vegetative growth stages.

Solar radiation and the bidirectional reflectance characteristics of vegetation canopies vary with the time of day and through the growing season (Ranson et al., 1985; Li et al., 2021), influenced by solar radiation, temperature, and shade levels. Pairwise comparison of the spectral information from bands and indices indicated no differences among different row directions for flights made after BBCH 61 and only a few differences for flights 3 to 9 at BBCH 17 to 61 (**Table 3**). In contrast, on the first two flights at BBCH 16 and 17, differences were frequently observed but primarily related to the east direction, which thus differed from the N, NE, and less often from the SE direction. The lower biomass observed in the eastern direction at early vegetative growth stages probably accounted for the spectral differences. Early in the growing season, because of low soil coverage, shade might influence the relationship between spectral indices and biomass (Chakhvasvili et al., 2022: Kuester and Spengler, 2018) or grain yield differently in various row compass directions, although such effects were not evident. This was also not observed in later growth stages because the spectral information from different row compass directions did not differ (**Table 3**), and the biomass coverage was high.

In agreement with previous studies, good relationships between spectral indices and biomass were found at vegetative stages (Mistele and Schmidhalter, 2008; Winterhalter et al., 2011) but also observed at the reproductive stages (Winterhalter et al., 2013). Grain yield was better predicted for averaged (mixed) row orientations than for single-row orientations, regardless of the angle of solar radiation. A high correlation of spectral indices with biomass and grain yield in mixed directions was observed from BBCH 55 onwards. There was no systematic influence of row orientation on the relationships between spectral data, biomass, and grain yield, except at the early growth stages.

Averaging over the entire season, particularly close relationships between the spectral indices GCI, GNDVI, and RECI were observed for the biomass assessment at BBCH 81 and grain yield at BBCH 99 for the N, NE, and E row directions, and more distant relationships were obtained for the SE direction (**Figure 4**). Other indices such as the NDREI, NDVI, and SR performed less well, except for increased values observed for the spectral relationship found in the NE direction and biomass. Although the indices GCI and GNDVI also best-predicted biomass until BBCH 73, they were overpassed by NDVI and SR after this growth stage.

Spectral relationships to biomass at BBCH 81 varied in row directions. They were very tight from the beginning of the experiment at BBCH 16 for the NE orientation, with R² values close to 0.9 for the indices NDVI, SR, GCI, and GNDVI. In contrast, the eastern orientation showed significantly fewer close relationships.

The grain yield was particularly well predicted by the indices GCI (**Figure 4**) and GNDVI and was consistently high after BBCH 61 and least close by the spectral index NDREI. The relationships were closer in individual compass directions, with R² values varying between 0.8–0.9, compared to those in mixed-row orientations with R^2^ = 0.7. We attribute the less close relationship in the model with mixed-row compass direction to variations in biomass growth found in the individual orientations.

Overall, grain yield was slightly better predicted than biomass, and predictions were generally close to or higher than R^2^ = 0.7 at BBCH 55, with a noticeable decrease in the morning flight at BBCH 61. It is noticeable from this study that grain yield did not differ in different compass directions; rather, small differences were observed in the vegetative biomass assessments at BBCH 17 and 61 in the east direction. In contrast to this study, Karlen and Kasperbauer (1989) found that at the growth stage, BBCH 71 plants grown in E-W rows yielded more than those grown in N-S rows at the growth stage BBCH 71 in 1985 and ascribed this to water stress effects.

Independent of the angle of solar radiation, the spectral indices were closely related to the biomass and grain yield of maize grown in the four compass directions. In contrast, the influence of crop row orientation has been demonstrated in optical wavelength domains through the use of bidirectional reflectance distribution functions concerning the viewing angle of satellite images (Kimes and Kirchner, 1983; Andrieu et al., 1997; Marais-Sicre et al., 2014). The differences can be related to the crop type and NADIR view of the drone sensing used in our study. In line with this, Li et al. (2021) reported that NDVI values of maize with and without tassels did not vary at UAV viewing angles of 30°, 45°, and 60° compared to NADIR at the reproductive stage, but crop-specific differences were observed for wheat and sunflower.

The sun’s position was likely to play a role only in the early growing season with little ground cover, as ANOVA revealed significant differences in the spectral values of individual row orientations after BBCH 17 in only a few cases. However, the spectral assessments of the biomass did not differ in the mixed-row directions.

Disregarding daytime changes at the same growth stages, the spectral values, as illustrated for the GCI, increased until BBCH 69 and then decreased (**Figure 6**). A closer inspection of daytime changes on given days indicated a diurnal trend with the lowest spectral values observed after midday. This observation agrees with the findings of Maresma et al. (2020), who, over two years, observed the lowest NDVI values between 11 am and 1 pm in maize at the V10 growth stage and ascribed this, particularly in one year, to the effects of water stress that provoked earlier curling in the maize leaves, resulting in a larger impact of the soil on the NDVI readings acquired close to solar noon. Thus, they obtained more accurate yield predictions at 9 am and 5 pm.

Changes in the fluorescence emission of light-harvesting pigments, which decrease with higher irradiation, can contribute to reflectance differences during the day (Hoel and Solhaug, 1998; Thoren et al., 2010; Campbell et al., 2019). In addition, changes in the water status (Elsayed et al., 2011; Rischbeck et al., 2014) and the leaf orientation during the day may contribute to the diurnal differences in reflectance.

The partially significant diurnal changes observed indicate the need to conduct measurements within a relatively narrow time window, which may be limited to one hour. The results of Maresma et al. (2020) highlighted the need for standardization of the timing of flights during the day. In addition, a longer measurement time will confound spectral comparisons in large fields or phenotyping in large breeding nurseries, requiring longer sensing. Overall, the results also indicate that measurements performed at different times cannot be compared with each other; thus, sensing performed at various sites and different times of day will probably not allow comparing the results with each other when, for example, evaluating the performance of different cultivars. Instead, a relative comparison should be made. Unless overcast weather conditions are not present, midday measurements are suggested as the best compromise for sensing with UAVs because radiation changes little during this time.

Our observations agree with a recent report that evaluated daytime changes with different terrestrial and UAV-based sensors and found that spectral indices differed significantly in wheat independent of the sensor platform used (De Souza et al., 2021). However, such differences may be more marked in other row crops because of varying solar angles.

Because nadir spectral measurements are still common for near-ground platforms owing to their simple implementation and because most satellite sensors at high spatial resolutions collect quasi-nadir data owing to the narrow field of view, such as 15° for the Operational Land Imager (Li et al., 2020) UAV-assisted sensing in highly dedicated field designs such as those used in this study could serve as a reference for ground-based and satellite-based reflectance sensing.

## Conclusions

Consistent diurnal and seasonal cycles of canopy reflectance of row-planted crops grown in different compass directions and their dependency on solar angle radiation have not been investigated and are reported in this study. Investigating the reflectance of plants grown in different row orientations requires a highly dedicated experimental design with comparable biomass and grain yields in different compass directions. Although some differences were observed in the earlier growth stages, this criterion was included in this study. Further evidence was provided by the pairwise spectral comparison of bands and indices, which indicated no differences in the different row orientations after BBCH 61, and only a few differences were observed in earlier vegetative stages. A close match between the spectral footprint and destructively assessed areas is necessary to investigate the spectral relationships with biomass and grain yield. The solar radiation angle did not affect spectral indices in the four compass directions. Except at the early growth stages in the vegetative stage, no systematic influence of row orientation on the relationships between spectral data and biomass and grain yield was observed. However, tighter relationships were obtained for the individual row orientations. Early in the growing season, good relationships were observed between spectral indices and biomass and grain yield; however, some differences were noted in specific row orientations. The spectral indices GCI, GNDVI, and RECI, were particularly well related to biomass and grain yield at BBCH 81 and BBCH 99, and other indices, such as NDREI, NDVI, and SR, performed less well. Generally, grain yield could already be predicted after BBCH 61 and was slightly better than that of biomass.Reflectance changes during the day and as crops grow during the season, and differences must be accounted for when establishing sensor-derived algorithms. Diurnal shifts in reflectance require the flight timing to be standardized. This must also be considered when comparing terrestrial, aerial, or satellite sensing information.

## Data availability statement

The raw data supporting the conclusions of this article will be made available by the authors, without undue reservation.

## Author contributions

CB curated and analyzed the data, wrote a first draft of the manuscript. US designed and conducted the experiment, assisted in data analysis, edited the publication, managed the project and acquired funds. All authors contributed to the article and approved the submitted version.

## Funding

This work was supported as part of the GreenWindows4_0 project by funds of the Federal Ministry of Food and Agriculture 516 (BMEL) based on a decision of the Parliament of the Federal Republic of Germany *via* the 517 Federal Office for Agriculture and Food (BLE) under the innovation support program.

## Conflict of interest

The authors declare that the research was conducted in the absence of any commercial or financial relationships that could be construed as a potential conflict of interest.

## Publisher’s note

All claims expressed in this article are solely those of the authors and do not necessarily represent those of their affiliated organizations, or those of the publisher, the editors and the reviewers. Any product that may be evaluated in this article, or claim that may be made by its manufacturer, is not guaranteed or endorsed by the publisher.
