# Supplementary material for: Daytime and seasonal reflectance of maize grown in varying compass directions
Source: Front Plant Sci. 2022 Sep 23;13:1029612. doi: 10.3389/fpls.2022.1029612 (PMC9539767; doi:10.3389/fpls.2022.1029612)
Supplement: Supplementary file 1 [file DataSheet_1.docx]

**Appendix**


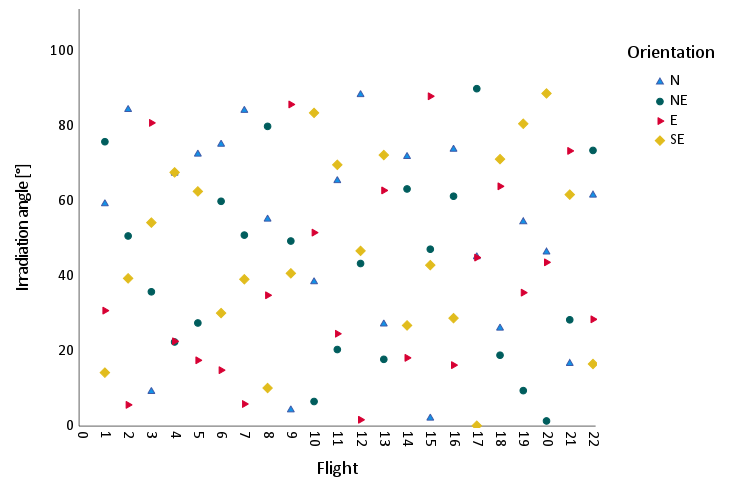


BBCH 61

BBCH 81

BBCH 17

Figure A1. Irradiation angles per row orientation at midday flight of the 22 UAV flights. The growth stages BBCH 17, 61, and 81, depicting repeated daytime measurements, are indicated in boxes.

Figure A2. Spectral relationships between information obtained in the grain yield area and the third, fourth, and fifth row are indicated as coefficients of determination. The rows were counted from the outside to the inside of the plot (cf. Figure 1).


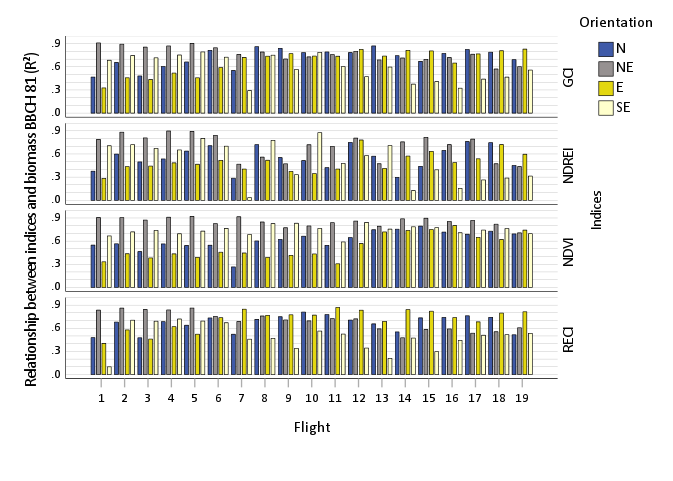


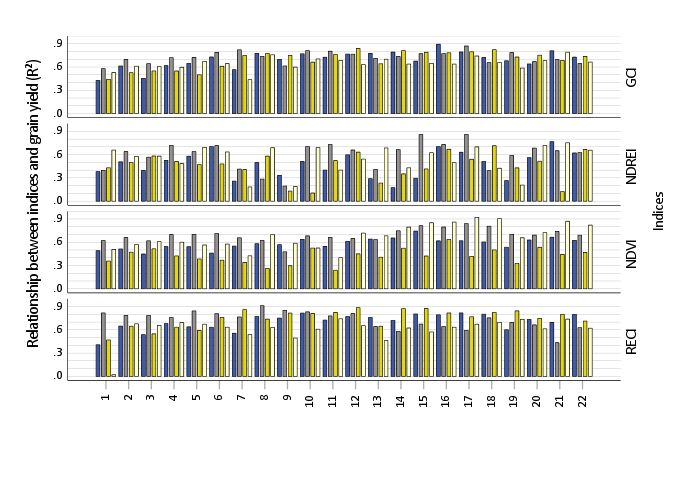


BBCH 17

BBCH 61

BBCH 81

Flight

BBCH 17

BBCH 61

BBCH 81

Flight

Figure A3. Relationships between the spectral indices GCI, NDREI, NDVI, RECI, and biomass and grain yield of maize grown in four compass directions at BBCH 81 and BBCH 99, respectively, as indicated by Pearson coefficients of determination for the 22 flights conducted across the growing season. BBCH growth stages of repeated daytime measurements are indicated in boxes.
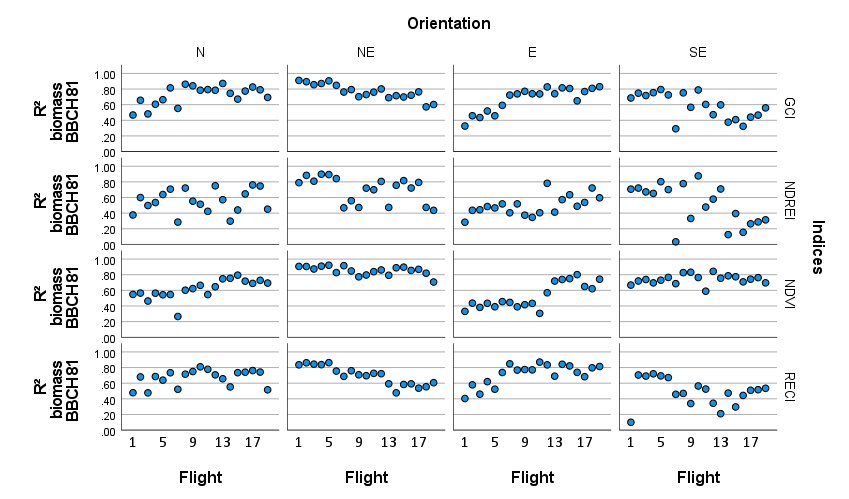


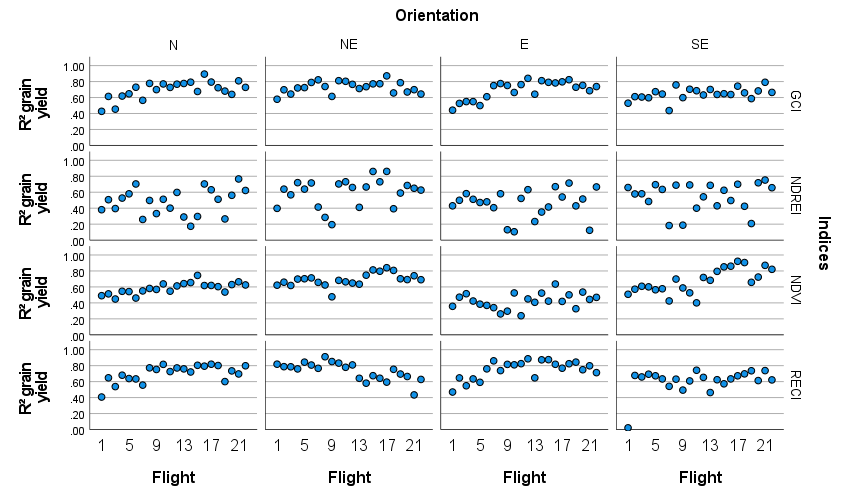


Figure A4. Spectral relationships of the indices GCI, NDREI, NDVI, and RECI to biomass and grain yield of maize grown in four compass directions at BBCH 81 and 99, respectively, are indicated by coefficients of determination (R^2^ values).

Table A1. Average standard deviation (SD) and range of yield at BBCH 17, 61, 81, and grain yield at BBCH 99 in t ha^-1^ indicated for four compass directions in which maize was grown.

| **Yield** | **Compass**  **direction** | **Average yield (t ha^.-1^)** | **SD of yield**  **(t ha^.-1^)** | **Range of yield (t ha^.-1^)** |
| --- | --- | --- | --- | --- |
| Biomass dry weight  at BBCH 17 | N | 3.1 | 0.6 | 2.7 |
|  | NE | 3.0 | 0.6 | 2.2 |
|  | E | 2.5 | 0.4 | 1.4 |
|  | SE | 2.8 | 0.6 | 2.2 |
| Biomass dry weight  at BBCH 61 | N | 10.2 | 1.2 | 4.8 |
|  | NE | 9.4 | 1.5 | 4.9 |
|  | E | 8.7 | 1.1 | 3.5 |
|  | SE | 9.3 | 1.5 | 5.1 |
| Biomass dry weight  at BBCH 81 | N | 18.6 | 2.7 | 10.1 |
|  | NE | 18.8 | 2.9 | 9.8 |
|  | E | 18.3 | 2.4 | 8.0 |
|  | SE | 17.7 | 3.6 | 13.4 |
| Grain dry weight  at BBCH 99 | N | 10.1 | 1.9 | 7.2 |
|  | NE | 10.1 | 1.8 | 6.9 |
|  | E | 10.3 | 1.9 | 6.7 |
|  | SE | 9.8 | 2.4 | 8.0 |

Table A2. Relationships between the destructively assessed biomass yield at BBCH 17 and 81, and grain yield, indicated as coefficients of determination (R²).

| **Compass direction** |  | **Biomass BBCH17** | **Biomass BBCH81** |
| --- | --- | --- | --- |
| N | Biomass BBCH 81 | 0.35 |  |
| NE | Biomass BBCH 81 | 0.51 |  |
| E | Biomass BBCH 81 | 0.12 |  |
| SE | Biomass BBCH 81 | 0.39 |  |
| N | Grain yield | 0.61 | 0.71 |
| NE | Grain yield | 0.35 | 0.69 |
| E | Grain yield | 0.38 | 0.80 |
| SE | Grain yield | 0.30 | 0.82 |

Table A3. Spectral relationships between bands and indices averaged across all flights and rows indicated as coefficients of determination (R²).

| **Bands and Indices** | **Green** | **Red** | **Red edge** | **NIR** | **GCI** | **GNDVI** | **NDREI** | **NDVI** | **RECI** |
| --- | --- | --- | --- | --- | --- | --- | --- | --- | --- |
| Red | 0.74 |  |  |  |  |  |  |  |  |
| Red edge | 0.73 | 0.44 |  |  |  |  |  |  |  |
| NIR | 0.12 | 0.19 | 0.09 |  |  |  |  |  |  |
| GCI | 0.84 | 0.71 | 0.46 | 0.31 |  |  |  |  |  |
| GNDVI | 0.85 | 0.71 | 0.46 | 0.32 | 0.98 |  |  |  |  |
| NDREI | 0.73 | 0.69 | 0.26 | 0.25 | 0.81 | 0.82 |  |  |  |
| NDVI | 0.59 | 0.83 | 0.25 | 0.43 | 0.79 | 0.79 | 0.72 |  |  |
| RECI | 0.72 | 0.56 | 0.58 | 0.29 | 0.86 | 0.85 | 0.50 | 0.64 |  |
| SR | 0.61 | 0.85 | 0.28 | 0.38 | 0.82 | 0.79 | 0.71 | 0.96 | 0.66 |

Table A4. Root mean square error values indicated in t ha^-1^ for the relationship between the spectral indices GCI, GNDVI, NDREI, RECI, SR, and grain yield at the respective flights and corresponding BBCH growth stages.

| Flight |  | | BBCH growth stage | | GCI | | GNDVI | | NDREI | | NDVI | | RECI | | SR | |
| --- | --- | --- | --- | --- | --- | --- | --- | --- | --- | --- | --- | --- | --- | --- | --- | --- |
| 1 | | 16 | | 1.54 | | 1.51 | | 1.54 | | 1.56 | | 1.72 | | 1.62 | |  |
| 2 | | 17 | | 1.62 | | 1.63 | | 1.76 | | 1.78 | | 1.37 | | 1.77 | |  |
| 3 | | 17 | | 1.43 | | 1.36 | | 1.50 | | 1.51 | | 1.30 | | 1.59 | |  |
| 4 | | 17 | | 1.36 | | 1.33 | | 1.48 | | 1.47 | | 1.21 | | 1.54 | |  |
| 5 | | 17 | | 1.31 | | 1.25 | | 1.39 | | 1.44 | | 1.18 | | 1.51 | |  |
| 6 | | 55 | | 1.24 | | 1.21 | | 1.42 | | 1.52 | | 1.14 | | 1.60 | |  |
| 7 | | 61 | | 1.36 | | 1.35 | | 1.85 | | 1.69 | | 1.20 | | 1.70 | |  |
| 8 | | 61 | | 1.02 | | 1.02 | | 1.44 | | 1.36 | | 1.04 | | 1.39 | |  |
| 9 | | 61 | | 1.33 | | 1.32 | | 1.89 | | 1.57 | | 1.15 | | 1.55 | |  |
| 10 | | 61 | | 1.09 | | 1.11 | | 1.50 | | 1.38 | | 1.04 | | 1.43 | |  |
| 11 | | 61 | | 1.07 | | 1.07 | | 1.47 | | 1.56 | | 1.02 | | 1.58 | |  |
| 12 | | 69 | | 1.06 | | 1.09 | | 1.32 | | 1.33 | | 1.00 | | 1.25 | |  |
| 13 | | 73 | | 1.14 | | 1.15 | | 1.58 | | 1.36 | | 1.30 | | 1.36 | |  |
| 14 | | 75 | | 1.10 | | 1.11 | | 1.66 | | 1.23 | | 1.20 | | 1.23 | |  |
| 15 | | 79 | | 1.11 | | 1.11 | | 1.39 | | 1.10 | | 1.11 | | 1.03 | |  |
| 16 | | 81 | | 1.04 | | 0.99 | | 1.27 | | 1.09 | | 1.12 | | 1.14 | |  |
| 17 | | 81 | | 0.94 | | 0.95 | | 1.16 | | 1.11 | | 1.13 | | 1.12 | |  |
| 18 | | 81 | | 1.14 | | 1.12 | | 1.51 | | 1.13 | | 1.02 | | 1.11 | |  |
| 19 | | 81 | | 1.15 | | 1.16 | | 1.67 | | 1.37 | | 1.15 | | 1.44 | |  |
| 20 | | 85 | | 1.20 | | 1.19 | | 1.33 | | 1.31 | | 1.17 | | 1.39 | |  |
| 21 | | 89 | | 1.07 | | 1.03 | | 1.42 | | 1.15 | | 1.29 | | 1.21 | |  |
| 22 | | 89 | | 1.17 | | 1.14 | | 1.25 | | 1.21 | | 1.19 | | 1.26 | |  |
